# Candida albicans Oropharyngeal Infection Is an Exception to Iron-Based Nutritional Immunity

**DOI:** 10.1128/mbio.00095-23

**Published:** 2023-03-13

**Authors:** Norma V. Solis, Rohan S. Wakade, Scott G. Filler, Damian J. Krysan

**Affiliations:** a Division of Infectious Diseases, Lundquist Institute for Biomedical Innovation at Harbor-UCLA Medical Center, Torrance, CA; b Department of Pediatrics, Carver College of Medicine, University of Iowa, Iowa City IA; c Department of Medicine, David Geffen School of Medicine at UCLA, Los Angles, CA; d Department of Microbiology and Immunology, Carver College of Medicine, University of Iowa, Iowa City, Iowa, USA; e Department of Molecular Physiology and Biophysics, Caver College of Medicine, University of Iowa, Iowa City IA; University of Texas Health Science Center

**Keywords:** *Candida albicans*, iron response, nutritional immunity, oropharyngeal candidiasis

## Abstract

Candida albicans is a commensal of the human gastrointestinal tract and a common cause of human fungal disease, including mucosal infections, such as oropharyngeal candidiasis and disseminated infections of the bloodstream and deep organs. We directly compared the *in vivo* transcriptional profile of C. albicans during oral infection and disseminated infection of the kidney to identify niche specific features. Overall, 97 genes were differentially expressed between the 2 infection sites. Virulence-associated genes, such as hyphae-specific transcripts, were expressed similarly in the 2 sites. Genes expressed during growth in a poor carbon source (*ACS1* and *PCK1*) were upregulated in oral tissue relative to kidney. Most strikingly, C. albicans in oral tissue shows the transcriptional hallmarks of an iron replete state while in the kidney it is in the expected iron starved state. Interestingly, C. albicans expresses genes associated with a low zinc environment in both niches. Consistent with these expression data, strains lacking transcription factors that regulate iron responsive genes (*SEF1*, *HAP5*) have no effect on virulence in a mouse model of oral candidiasis. During microbial infection, the host sequesters iron, zinc, and other metal nutrients to suppress growth of the pathogen in a process called nutritional immunity. Our results indicate that C. albicans is subject to iron and zinc nutritional immunity during disseminated infection but not to iron nutritional immunity during oral infection.

## OBSERVATION

During microbial infection, mammalian hosts sequester metal micronutrients to reduce replication of infecting organisms. This strategy is referred to as nutritional immunity, and is an important feature of the host response to a variety of microbial pathogens (1). For example, the human fungal pathogen Candida albicans shows the transcriptional signature of iron starvation during disseminated infection of the kidney (2, 3). Furthermore, C. albicans strains lacking genes required for survival in low iron environments are less virulent in mouse models of disseminated infection (4). C. albicans is also a commensal of the human oral cavity and gastrointestinal tract. In its commensal state within the gut, C. albicans appears relatively iron replete based on the expression of iron-regulated transcription factor (TF) genes, such as *SEF1* and *SUF1*, and their target genes (4). Thus, the commensal GI niche C. albicans is inferred to be iron replete, while invasion of the kidney places the fungus in an iron-deficient state.

In addition to being a commensal of the oral cavity, C. albicans can also invade the local submucosae to cause oropharyngeal candidiasis (OPC) in susceptible patients, including those with reduced T-cell function, living with HIV/AIDS, or undergoing oral radiation therapy (5). The oral submucosa is anatomically and physiologically distinct from target organs of disseminated candidiasis, such as kidney, liver, and spleen (6). To characterize the effect of different infection environments on the transcriptional profiles of C. albicans, we performed *in vivo* transcriptional profiling using Nanostring nCounter technology and a set of environmentally responsive genes during infection of either mouse kidney or tongue using standard models of disseminated (2) and oropharyngeal candidiasis (6, 7), respectively.

**FIG 1:**
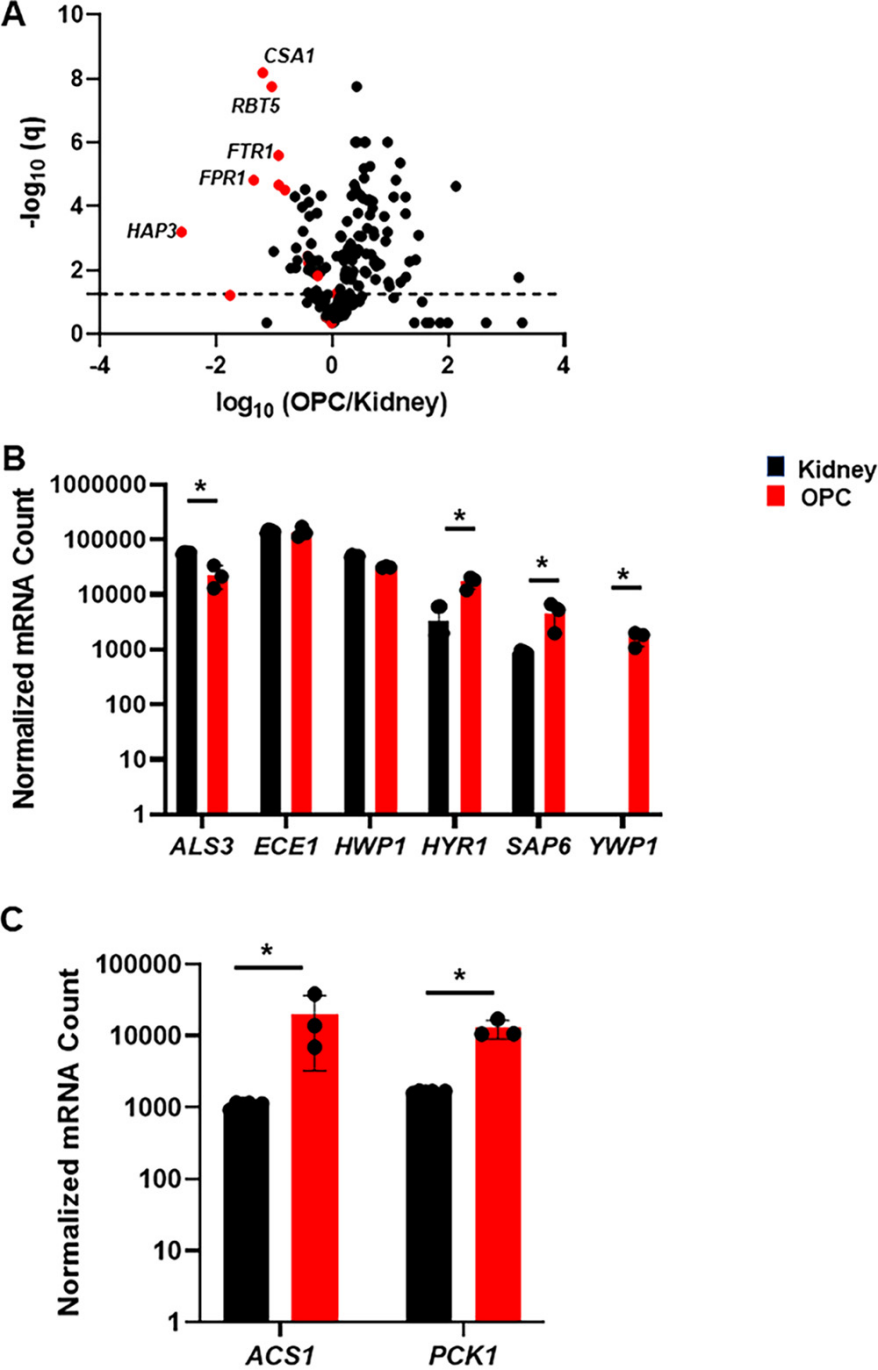
The expression of environmentally responsive and virulence genes is distinct during oral and disseminated kidney infection models. (A) The volcano plot shows log_10_ fold change of genes (OPC/kidney) with the false discovery rate q (Benjamini-Yekutieli) cutoff 0.1 shown by the horizontal line on the plot. Genes that are induced in low Fe conditions are highlighted in red. (B) The normalized expression of hyphae-specific genes (A*LS3*, *ECE1*, *HWP1*, and *SAP6*) and a yeast specific gene (*YWP1*) are shown in log_10_ scale. *YWP1* was undetectable in the kidney samples. Asterisk * indicates adjusted *P* value (q) < 0.1. (C) Genes indicative of glucose starvation, *ACS1* and *PCK1*, are induced in oral tissue relative to kidney tissue.

Overall, 97 genes were differentially expressed when OPC is compared to kidney; 30 genes downregulated and 67 upregulated (±2 fold with FDR < 0.1; Benjamini-Yekutieli) as summarized in the volcano plot (Fig. 1A and See Table S1 for raw data, data normalization, and analysis). C. albicans is a dimorphic fungus that exists primarily in the hyphal state when infecting either kidney or oral tissue (8). Virulence-associated, hyphae-specific genes, such as *ECE1*, *ALS3*, and *HWP1* were expressed comparably in the 2 infection sites, while *HYR1* and *SAP6* were expressed higher in oral tissue (Fig. 1B). Thus, the expression profiles indicate that C. albicans is primarily in the invasive, hyphal morphology in both infection sites. Interestingly, expression of the yeast phase specific gene *YWP1* (9) is undetectable in the kidney but is expressed in the oral cavity. Although the expression of *YWP1* is 30-fold lower than that observed under yeast phase growth *in vitro* (10), this indicates that the yeast phase may be more prevalent in OPC compared to the kidney.

10.1128/mbio.00095-23.2 TABLE S1 Expression of environmentally responsive C. albicans genes in kidney during disseminated infection and tongue from oropharyngeal infection. The raw counts for kidney (6replicates) and tongue (3 replicates), normalized counts, mean, standard deviation, fold change OPC relative to kidney, *P* value (Student t test), and adjusted *P* value (q) from Benjamini-Yekutieli procedure are shown. Green are genes upregulated 2-fold and red are genes downregulated 2-fold with false discovery rate (q) < 0.1. Download Table S1, XLSX file, 0.07 MB. Copyright © 2023 Solis et al. 2023 Solis et al. https://creativecommons.org/licenses/by/4.0/ This content is distributed under the terms of the Creative Commons Attribution 4.0 International license.

These transcriptional data also provide insights into the relative carbon and metal nutrient status of C. albicans at the 2 infection sites. First, *PCK1* and *ACS1*, enzymes that mediate gluconeogenesis (*PCK1*) and non-glucose-derived acetyl-CoA synthesis (*ACS1*), are highly expressed in oral tissue relative to the kidney (Fig. 1C). *In vitro*, *PCK1* (11) and *ACS1* (12) are suppressed by glucose and induced by poor carbon sources, such as lactate or glycerol. As such, C. albicans appears more dependent on non-glucose carbon sources when infecting oral tissue relative to kidney tissue. Second, zinc is a critical micronutrient that is sequestered by the host in response to disseminated C. albicans infection (2, 13), leading to expression of C. albicans genes indicative of zinc starvation. C. albicans infecting oral tissue expresses zinc-related genes at statistically similar levels to kidney (Fig. S1).

10.1128/mbio.00095-23.1 FIG S1 Genes involved in zinc starvation are expressed similarly in C. albicans infecting oral and kidney tissue. The expression of zinc-responsive genes is similar in oral and kidney tissue. Asterisk * indicates adjusted *P* value (q) < 0.1. Raw, processed, and analyzed data are available in Table S1. Download FIG S1, TIF file, 0.2 MB. Copyright © 2023 Solis et al. 2023 Solis et al. https://creativecommons.org/licenses/by/4.0/ This content is distributed under the terms of the Creative Commons Attribution 4.0 International license.

**FIG 2:**
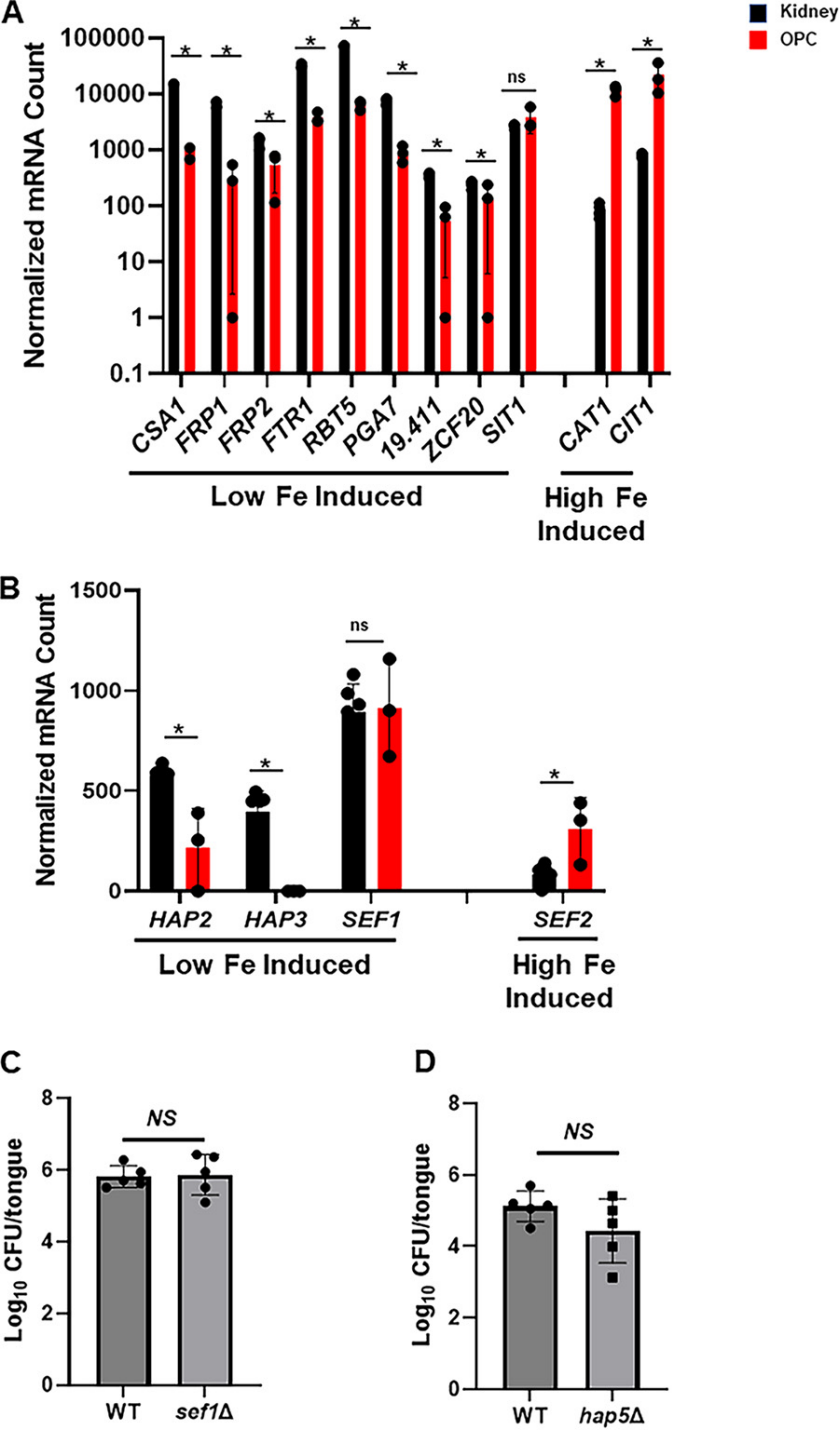
C. albicans does not show characteristics of iron starvation during oral infection. (A) A set of 11 non-regulatory genes that are regulated in response to iron were in our probe set. The bars indicate normalized RNA levels determined by Nanostring for kidney derived samples (*n* = 6) and oral tissue derived samples (*n* = 3). Asterisk * indicates adjusted *P* value (q) < 0.1. (B) Four iron responsive transcription factor genes were also in the set and their expression is shown. The oral fungal burden (CFU/tongue) at day 5 postinfection is shown for 2 transcriptional regulators of the response to iron starvation *SEF1* (C) and *HAP5* (D). The fungal burden in mice infected with the 2 mutants did not differ from that of mice infected with the WT reference strain (Student's *t* test, *P* > 0.05).

Third, 15 genes in our set are iron responsive and controlled by a well-characterized transcription factor circuit (4, 14). Of the non-regulatory genes induced by iron starvation, 8/9 are expressed higher in kidney tissue relative to oral tissue (Fig. 2A), while both genes in our set that repressed by iron starvation were expressed higher in oral tissue. Consistent with this transcriptional signature (4, 14), the expression of 3 of 4 TFs that regulate the expression of genes during iron-deficiency (*HAP2*, *HAP3*, *and HAP43*; fold change for *HAP43* was 0.51 and was statistically significant q = 0.013) were expressed lower in C. albicans infecting oral tissue relative to kidney. The exception is that *SEF1*, a TF downregulated *in vitro* by iron depletion, is expressed similarly in both sites. *SEF2* is expressed higher under iron replete conditions and is expressed higher in oral tissue relative to kidney (Fig. 2C). Overall, 13/15 iron responsive genes are expressed in a pattern consistent with C. albicans being in an iron replete state in oral tissue, and an iron starvation state in kidney tissue.

If these transcriptional distinctions have pathobiological significance, then deletion of TFs required for replication in low iron environments should have no effect on OPC virulence. To test this hypothesis, we examined the virulence of strains lacking *SEF1* and *HAP5*, two TFs required for *in vitro* growth of C. albicans on low iron media. We chose these 2 mutants because *SEF1* functions in the initiation of the transcriptional response to iron starvation, while *HAP5* is a downstream regulator of the response (4). The oral fungal burden of mice infected with either the *sef1*ΔΔ or *hap5*ΔΔ mutants was not different than WT (Fig. 2D). Thus, low iron response transcriptional regulators are dispensable for C. albicans virulence in OPC. These genetic results are consistent with our transcriptional data and provide strong support for a model in which C. albicans in oral tissue is not under iron starvation.

Taken together, these data suggest that C. albicans infection of the kidney appears to induce iron nutritional immunity while that of the oral cavity does not. Nutritional immunity is mediated by innate immune cells and non-immune cells, such as epithelial cells (2, 15); thus, it is likely that differences in iron nutritional immunity in the 2 niches are multifactorial. For example, iron is the second most abundant metal in saliva and a significant proportion of the total iron in saliva is soluble (~ 30%), indicating there may be a substantial pool of iron available in the oral tissue (16, 17). In addition, cells of the oral mucosae lack divalent cation transporters present in other mucosal tissues and may be less able to import extracellular iron (18). As such, it is possible that C. albicans more effectively scavenges iron in oral tissue compared to kidney.

Immunocompetent mice rapidly clear C. albicans from oral tissue and immunosuppression either by steroid treatment (as used here) or deletion of IL-17, a key regulator of host immunity to mucosal C. albicans infection, is required to establish infection (7). Since the innate immune system also participates in nutritional immunity through the actions of macrophages and neutrophils and related cytokines, such as TNF-α, IL-1, and IL-6 (15), we cannot rule out the possibility that immune suppression may partially blunt nutritional immunity. However, because zinc nutritional immunity is dependent on the same innate immune responses as iron, we would expect both iron and zinc responses to be blunted in the oral cavity if phagocyte sequestration of micronutrients was the primary mechanism for the distinction in iron status of C. albicans between the 2 infection sites; this was not the case. Further work will be required to dissect the mechanism of these observations. Regardless of the mechanisms, these highlight how variations in the local physiological environment of host niches impact not only pathogen physiology but also the nature of the host response and the virulence traits expressed by the pathogen.

### General methods and strains.

All C. albicans strains were in the SN background and have been previously reported (19). The low iron growth phenotypes for the *sef1*ΔΔ and *hap5*ΔΔ mutants were confirmed. Yeast strains were struck from frozen stocks and pre-cultured in yeast peptone dextrose medium at 30°C prior to preparation of inoculum for infection.

### Oropharyngeal candidiasis model.

The immunosuppressed mouse model of OPC was employed, as previously described with some modifications (7). Male ICR mice were injected subcutaneously with cortisone acetate (300 mg/kg of body weight) on infection days: −1, 1, and 3. On the day of infection, the animals were sedated with ketamine and xylazine and a swab saturated with C. albicans strain SN250, the *sef1*ΔΔ mutant, or the *hap5*ΔΔ mutant (10^6^ cells per mL) was placed sublingually for 75 min. On postinfection day 5, the mice were sacrificed, and the tongues were harvested. For fungal burden studies, the harvested tongues were homogenized and plated for quantitative fungal burden (*n* = 5 per strain). The log_10_-transformed fungal burden data for each experiment was analyzed by Student's *t* test to identify statistically significant differences between individual strains (*P < *0.05). For expression studies, mice were sacrificed after 5 days of infection, and the tongues were harvested. Using a cell scraper, the C. albicans was scrapped off the tongue, and RNA was extracted from the collected cells according to the manufacturer protocol (RiboPure RNA purification kit).

### Disseminated candidiasis model.

As previously described (10), 5 to 6 weeks old, female DBA2/N mice (Envigo) were inoculated with 5 × 10^4^ CFU of SN250 by lateral tail vein injection. After 48 h, mice were euthanized, kidneys harvested, and placed directly into ice-cold RNA Later solution (*n* = 6). The kidneys were then flash frozen in liquid nitrogen and ground into a fine powder with liquid nitrogen. The resulting tissue powder was mixed with ice-cold TRIzol. The samples were placed on a rocker at room temperature (RT) for 15 min, and the cell debris were removed by centrifuged the samples at 10K rpm at 4°C for 10 min. Cleared TRIzol was collected into a new 1.5 mL Eppendorf tube and 200 μL of RNase free chloroform was added. Tubes were shaken vigorously for 15 s and kept at RT for 5 min. Further, the samples were centrifuged at 12K rpm for 15 min at 4°C. The cleared aqueous layer was then transferred to new 1.5 mL tube, and RNA was further extracted following the Qiagen RNeasy kit protocol.

### Nanostring analysis.

As previously reported (10) and following standard protocols for *in vivo* Nanostring analysis (20), total RNA isolated from infected kidney and tongue (40 ng for kidney sample and 3 μg tongue sample). The differences in total RNA used reflects differential fungal burden and efficiency of fungal RNA isolation. The amount of C. albicans RNA in the tissue extracts was estimated using quantitative PCR for the *ACT1* gene and a standard curve. The indicated amounts of total RNA were added to a NanoString codeset mix (Table S1) and incubated at 65°C for 18 h. After hybridization reaction, samples were proceeded to nCounter prep station and samples were scanned on an nCounter digital analyzer. NCounter .RCC files for each sample were imported into nSolver software to evaluate the quality control metrics. Using the negative control probes, the background values were first assessed. The mean plus standard deviation of negative control probes value was defined and used as a background threshold, and this value is subtracted from the raw counts. The background subtracted total raw RNA counts were normalized against the highest total counts from the biological replicates to account for differences in fungal burden in the different samples. With the exception of 1 sample, the fungal RNA counts varied by less than 2-fold.

The statistical significance of changes in RNA counts was determined by two-tailed Student's *t* test (*p* < 0.05), followed by correction for multiple comparisons using the Benjamini-Yekutieli procedure and a false discovery rate or q value of 0.1. The expression data are summarized in Table S1. Probes that were below background were set to a value of 1 to allow statistical analysis. The raw counts, normalized counts, and statistical analyses are also provided in Table S1. The expression of 2 housekeeping genes (*ARP3* and *YRA1*) were used for normalization of C. albicans Nanostring data as reported by Huang et al. (21), and included in our probe set, varied by less than 20% between the 2 infections sites: fold change OPC/Disseminated: *ARP3*: 0.81; *YRA1* 0.82. A third housekeeping gene *CMK2* had a fold change of 1.1 between the 2 data sets. Please see Table S1 for raw and processed data. The data for the kidney infection model was previously reported (10). The 15 genes in our set that were identified as iron responsive were known to be regulated by the iron transcriptional responsive network in Chen et al. (4).
