# Supplementary figures and images for: Candida albicans Oropharyngeal Infection Is an Exception to Iron-Based Nutritional Immunity
Source: mBio. 2023 Mar 13;14(2):e00095-23. doi: 10.1128/mbio.00095-23 (PMC10128012; doi:10.1128/mbio.00095-23)

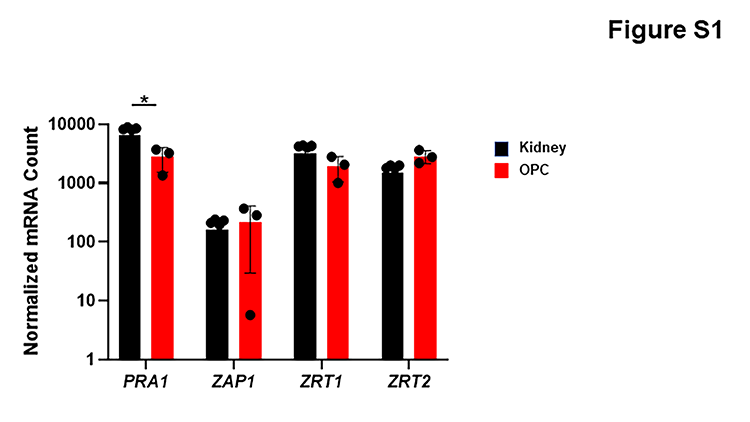

Supplement: FIG S1 [file mbio.00095-23-s0001.tif]
